# Routine management, healthcare resource use and patient and carer‐reported outcomes of patients with transfusion‐dependent β‐thalassaemia in the United Kingdom: A mixed methods observational study

**DOI:** 10.1002/jha2.282

**Published:** 2021-09-08

**Authors:** Farrukh Shah, Paul Telfer, Mark Velangi, Shivan Pancham, Robert Wynn, Sally Pollard, Elizabeth Chalmers, Jonathan Kell, Angela M. Carter, Joe Hickey, Clark Paramore, Minesh Jobanputra, Kate Ryan

**Affiliations:** ^1^ Whittington Hospital London UK; ^2^ Centre for Genomics and Child Health Blizard Institute Queen Mary University of London London UK; ^3^ Birmingham Children's Hospital Birmingham UK; ^4^ Birmingham City Hospital Birmingham UK; ^5^ Royal Manchester Children's Hospital Manchester UK; ^6^ Bradford Royal Infirmary Bradford UK; ^7^ Royal Hospital for Children Glasgow UK; ^8^ University Hospital of Wales Cardiff UK; ^9^ OPEN Health Marlow UK; ^10^ bluebird bio Cambridge Massachusetts USA; ^11^ bluebird bio UK Basingstoke UK; ^12^ Manchester Royal Infirmary Manchester UK

**Keywords:** blood transfusion, healthcare resource use, iron chelation therapy, quality of life, transfusion‐dependent β‐thalassaemia

## Abstract

**Objectives:**

We evaluated routine healthcare management, clinical status and patient‐ and carer‐reported outcomes in UK paediatric and adult patients with transfusion‐dependent β‐thalassaemia (TDT).

**Methods:**

A multi‐centre, observational mixed‐methodology study evaluated 165 patients (50% male; median age 24.1 [interquartile range (IQR)] 11.8–37.2] years) from nine UK centres.

**Results:**

Patients had a mean of 13.7 (standard deviation [SD] ±3.2) transfusion episodes/year (mean retrospective observation period 4.7 [±0.7] years). The median (IQR) for iron overload parameters at the last assessment during the observation period were: serum ferritin (*n* = 165) 1961.0 (1090.0–3003.0) μg/L (38% > 2500 μg/L); R2 liver iron (*n* = 119) 5.4 (2.9–11.6) mg/g (16% ≥15 mg/g); T2* cardiac iron (*n* = 132) 30.3 (22.0–37.1) ms (10% < 10 ms). All patients received ≥1 iron chelator during the observation period; 21% received combination therapy. Patients had a mean of 7.8 (±8.1) non‐transfusion‐related hospital attendances or admissions/year. Adult patients’ mean EQ‐5D utility score was 0.69 (±0.33; *n* = 94 [≥16 years]) and mean Transfusion‐dependent quality of life score was 58.6 (±18.4; *n* = 94 [≥18 years]). For Work Productivity and Activity impairment, mean activity impairment for patients ≥18 years (*n* = 88) was 48% (±32%) and for carers (*n* = 29) was 28% (±23%).

**Conclusions:**

TDT presents significant burden on patients, carers and healthcare resources.

## 1 INTRODUCTION

Transfusion‐dependent β‐thalassaemia (TDT) is a rare, severe genetic disease affecting adult haemoglobin production, necessitating lifelong blood transfusions and iron chelation therapy (ICT) [1, 2, 3]. Blood transfusions lead to iron overload requiring regular monitoring and optimisation of ICT to mitigate complications; however, this intensive therapy and monitoring imposes a great burden on patients and their families [4, 5, 6, 7, 8]. Complications associated with iron overload include endocrine dysfunction and hepatic pathology; cardiac disease is the most serious, leading to arrhythmias and heart failure, and early mortality [1, 3, 9]. While life expectancy for patients with TDT has significantly improved in the past 50 years in the United Kingdom (UK) [10], the crude 10‐year mortality rate for patients with TDT in England was recently reported as more than five times higher than the age/sex adjusted general population (6.2% vs. 1.2%; *p* < 0.001) [11], suggesting that despite care delivered in a high‐income setting, mortality from TDT remains significantly higher than the general population.

Until recently, allogeneic haematopoietic stem cell transplantation (allo‐HSCT) was the only potentially curative treatment. Allo‐HSCT is generally reserved for paediatric patients with a human leukocyte antigen‐matched related donor (25–30% of patients [12]), and not routinely offered for UK adult patients [3]. Recent advances in gene therapy have increased the avenues for potentially transformative treatments for TDT [13]. An understanding of the routine management of TDT and associated burden on patients, carers and healthcare resources is important in evaluating the impact of emerging treatments.

The present study evaluated current management pathways for TDT, clinical status, healthcare resource use, and the impact of TDT on quality of life (QoL) and work productivity of patients and carers in the United Kingdom.

**Plain language summary**

1. **What is the new aspect of your work?**

We report the results of a mixed methods observational study that provides an integrated understanding of the clinical status of a contemporary group of patients with transfusion‐dependent β‐thalassaemia (TDT; an inherited blood disorder requiring lifelong, regular blood transfusions along with management of iron introduced by these transfusions), quality‐of‐life data from affected patients and their carers and the impact of the condition on secondary healthcare services in the United Kingdom (UK).

1. **What is the central finding of your work?**

Patients with TDT in the study had significant transfusion burden, suffered a range of associated medical conditions and a subset were found to have moderate/high iron levels in the liver and heart, the latter of which can lead to early death. Quality of life of patients and carers and patients’ work productivity were impaired. Marked impact on secondary care services was also demonstrated.

1. **What is (or could be) the specific clinical relevance of your work?**

Despite management in expert centres, some patients with TDT require more aggressive management to reduce the risk of morbidity and early mortality. New therapeutic options that expand treatment choices beyond regular blood transfusions and iron management may improve outcomes. Healthcare providers may consider reconfiguration of secondary care services to support patient care and optimise use of UK National Health Service resources. Employers should be aware of the impact of TDT and its treatment on affected patients.

## 2 METHODS

### 2.1 Study design and setting

**FIGURE 1:**
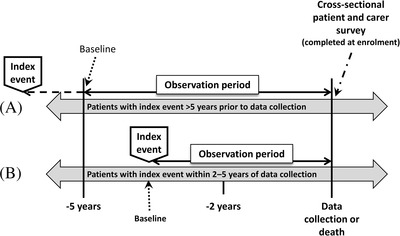
Study design. The index event was diagnosis of transfusion‐dependent β‐thalassaemia (TDT). For the purposes of this study, TDT (or TDT recurrence post‐allogeneic haematopoietic stem cell transplantation) was defined as β‐thalassaemia treated with a minimum of eight transfusion episodes during the first year of chronic transfusion therapy or a history of at least 100 mL/kg/year of red blood cells. The study observation period for eligible patients with TDT diagnosed ≥ 5 years prior to data collection was the 5‐year period prior to data collection or death (A). For eligible patients with TDT diagnosed 2–5 years prior to data collection, it was the period from TDT diagnosis to data collection or death (B). Baseline was defined as the start of the patient's observation period

A multi‐centre, observational mixed‐methodology study, involving a retrospective chart review and cross‐sectional survey of paediatric and adult patients with TDT and their carers, was conducted in nine UK National Health Service (NHS) centres. The study observation period for eligible patients with TDT diagnosed ≥5 years prior to data collection was the 5‐year period prior to data collection or death; for patients with TDT diagnosed 2–5 years prior to data collection it was the period from TDT diagnosis to data collection or death (Figure 1). Baseline was defined as the start of the patient's observation period. Centres were selected in order to give a good geographical spread (including centres in England, Scotland and Wales) and to be representative of practice across the range of centres managing patients with TDT in the UK, including adult haematology centres, paediatric only centres and centres managing both adult and paediatric patients. Potential centres were identified and selected based on a feasibility questionnaire and meeting; with the number of potentially eligible patients in the selected centres ranging between 5 and 53.

### 2.2 Participants

#### 2.2.1 Patients with TDT

Patients with TDT or with TDT recurrence after allo‐HSCT ≥2 years prior to data collection were eligible for inclusion. TDT, or TDT recurrence post‐allo‐HSCT, was defined as follows: β‐thalassaemia treated with a minimum of eight transfusion episodes during the first year of chronic transfusion therapy (or the first year of chronic transfusion therapy after recurrence post‐allo‐HSCT) or transfusion of at least 100 mL/kg/year of red blood cells.

For the retrospective chart review, exclusion criteria included patients who remained TDT‐free post‐allo‐HSCT, patients with <2 years of continuous data prior to data collection, and patients participating in any clinical trial during the observation period. For the optional cross‐sectional survey, exclusion criteria included patients with any significant mental or English language incapacity preventing them from participating.

#### 2.2.2 Carers of patients with TDT

The carers (parents or carers) of patients completing the patient survey were eligible for the carer survey if they were aged ≥16 years and had no significant mental or English language incapacity preventing them from participating.

#### 2.2.3 Patient and carer consent

Living patients (or their carer, as appropriate for age), and carers participating in the survey provided written informed consent according to the protocol approved by the NHS Heath Research Authority (London–Harrow Research Ethics Committee reference: 17/LO/2114; 14 March 2018). For deceased patients, data were collected by members of the direct care team to preserve patient confidentiality.

### 2.3 Endpoints and data collection

The primary endpoint was the number of blood transfusion episodes/patient/year. Secondary endpoints included: metrics of routine management of TDT (including transfusions and ICT); patients’ clinical status, TDT treatment‐related complications and comorbidities (as documented in medical records); TDT‐related healthcare resource utilisation (including hospital attendances, admissions and iron overload assessments); and patient and carer QoL. For the retrospective chart review, anonymised data were collected from written and electronic hospital record systems using standard electronic data collection forms designed specifically for the study. All staff involved in data collection were trained in the requirements for the study documentation. Data were checked for completeness and accuracy using manual and programmed validation checks and queries resolved through discussion with centre investigators. Data collection was undertaken between June 2018 and July 2019.

Patients and carers completed three validated questionnaires (using anonymised paper‐based questionnaires) evaluating different aspects of QoL and work productivity, using age‐appropriate and carer‐specific versions where available (see Supporting Information Supplementary Table S1). General health status was evaluated using the generic EuroQoL EQ‐5D 3 level (EQ‐5D‐3L) questionnaire [14, 15, 16]. TDT‐specific QoL was evaluated using the Transfusion‐dependent QoL (TranQoL) questionnaire [17, 18]. TDT impact on work and daily activities was evaluated using the Work Productivity Activity Index (WPAI) questionnaire [19, 20, 21].

The EQ‐5D‐3L questionnaire was used (under licence) to assess the general health status of patients and their carers on the day of completion. Adults (≥16 years) were administered the EQ‐5D‐3L and children (8–15 years) were administered the EQ‐5D‐Y, a child‐friendly EQ‐5D version. A proxy version of the EQ‐5D‐Y was administered only to carers of children between the ages of 4–7 years, which required the proxy to rate the health of the child. No version of the EQ‐5D‐Y is available for children aged under 4 years [14, 15, 16]. The EQ‐5D‐3L comprised two parts. The first evaluated five dimensions of health (mobility, self‐care, usual activities, pain/discomfort and anxiety/depression) on three levels (level 1: no problems; level 2: some problems; level 3: extreme problems) to give a 5‐digit health state that was converted to a summary health utility score ranging from 1 (perfect health) to < 0 (0 is equivalent to death and negative values represent states worse than death) using the UK value set [22]. There is currently no value set for the self‐completed EQ‐5D‐Y (8–15 years); therefore, utility scores could not be calculated for this age group [14]. The second part of the EQ‐5D‐3L used a visual analogue scale (VAS) to assess self‐rated health from 0 (worst imaginable) to 100 (best imaginable). The EQ‐5D‐Y measured the same dimensions as the EQ‐5D‐3L worded in a manner suitable for completion by children.

TDT‐related QoL for patients and carers was assessed using the TranQoL questionnaire (under a user agreement). Adults (≥18 years) were administered the adult version, carers were administered the parent version to complete about themselves, children (7–17 years) were administered the child version. A proxy version of TranQoL was administered only to carers of children <7 years of age [17, 18]. TranQoL is a thalassaemia‐specific QoL instrument that assesses the impact of disease over the 7 days prior to completion on physical health, emotional functioning, family functioning and school and career functioning, with the adult version also covering sexual activity. The individual domain scores and the TranQoL overall score range from 0 to 100, with higher scores representing better QoL[18].

The impact of TDT was also assessed using the WPAI questionnaire for adult patients (≥18 years) and carers only; the WPAI specific health problems (SHP) version for patients and the caregiver (WPAI:CG) version for carers were used as appropriate [19, 20, 21]. The WPAI assesses the impact of disease on work productivity over the 7 days prior to completion by evaluating absenteeism (the amount of work time missed), presenteeism (impaired working effectiveness [WPAI definition]) and overall work productivity loss (absenteeism and presenteeism), as well as assessing activity impairment (impact on non‐work related activities). Domain scores are expressed as percent impairment, with higher scores indicating a greater degree of impairment [19, 20].

### 2.4 Statistical analyses

Normally distributed quantitative variables and variables describing hospital resource utilisation are presented as mean (± standard deviation). Non‐normally distributed quantitative variables are presented as medians (interquartile range [IQR]). Distributions are presented where appropriate. Categorical variables are described as frequency (percentage). Annualised variables were estimated after excluding periods with missing data. Results for haemoglobin tests were batch‐exported; haemoglobin assessments were classified as pre‐transfusion haemoglobin assessments if they occurred within 3 days prior to a transfusion episode.

*Post hoc* analyses were conducted to evaluate the relationships between selected endpoints and cut‐off points for iron burden based on UK Thalassaemia Society Standards for serum ferritin (low serum ferritin: <500 μg/L; recommended serum ferritin: 500–1000 μg/L; moderate serum ferritin: >1000 to ≤2500 μg/L; high serum ferritin: >2500 μg/L), liver iron R2 concentration (low liver iron: <3 mg/g; recommended liver iron: 3–7 mg/g; moderate liver iron: >7 to <15 mg/g; high liver iron: ≥15 mg/g) and cardiac iron T2*concentration (normal cardiac iron: >20 ms; moderate cardiac iron: 10–20 ms; severe cardiac iron: <10 ms; very severe cardiac iron: <6 ms) [3].

Percentages are rounded to the nearest whole number (hence may not sum to 100%). All analyses were conducted using only available results and denominators presented where data were missing.

## 3 RESULTS

### 3.1 Patient demographics and clinical characteristics

Patient demographics and clinical characteristics are summarised in Table 1. The study included 165 patients with TDT (median age 24.1 [IQR 11.8–37.2] years, 25% aged <12 years; 50% male; 70% were of Asian origin, that is Pakistani, Bangladeshi, Indian or mixed Asian origin; two patients had recurrence of TDT after allo‐HSCT; one patient was deceased). Of 156 patients with baseline comorbidity data, 69% had ≥1comorbidity (most commonly hypogonadotropic hypogonadism [20%], splenectomy [20%], cardiac disease [17%] and vitamin D deficiency [16%]).

**TABLE 1:** Patient demographics and clinical characteristics

Demographic and clinical characteristics	Patients with TDT (*n* = 165)
Age at the end of the observation period (years), median (IQR)	24.1 (11.8–37.2)
Age distribution, *n* (%)	
<12	42 (25%)
12 < 18	19 (12%)
18 < 30	42 (25%)
30 < 40	29 (18%)
40 < 50	18 (11%)
50 < 60	12 (7%)
≥60	3 (2%)
Male, *n* (%)	82 (50%)
Ethnicity, *n* (%)	n = 148
Pakistani	49 (33%)
Bangladeshi	18 (12%)
Indian	17 (11%)
Chinese	7 (5%)
White and Asian	1 (1%)
Any other Asian background	19 (13%)
White and Black African	1 (1%)
Any other White background	28 (19%)
Any other Black background	1 (1%)
Any other Mixed background	3 (2%)
Other ethnic group	4 (3%)
Not stated	17
Comorbidities at baseline	n = 156
None	49 (31%)
≥1	107 (69%)
Not known	9
Comorbidities recorded in ≥5 patients, n (% of 156)	
Hypogonadotropic hypogonadism^a^	31 (20%)
Splenectomy^a^	31 (20%)
Cardiac disease^a^	26 (17%)
Vitamin D Deficiency	25 (16%)
Osteoporosis^a^	22 (14%)
Diabetes^a^	20 (13%)
Hepatitis^a^	16 (10%)
Osteopenia^a^	13 (8%)
Hypothyroidism^a^	12 (8%)
Asthma	11 (7%)
Hypogonadism^a^	11 (7%)
Growth failure^a^	9 (6%)
Eczema	8 (5%)
Liver iron overload^a^	7 (4%)
Impaired glucose tolerance^a^	7 (4%)
Hypoparathyroidism^a^	6 (4%)
Other	81
Duration of TDT at baseline (years), median (IQR)	9.5 (3.6–18.5), *n* = 112
Age at TDT diagnosis (years), median (IQR)	1.0 (0.5–8.0), *n* = 107

^a^ Comorbidities likely to be TDT‐related. IQR: interquartile range. TDT: transfusion‐dependent β‐thalassaemia.

### 3.2 Transfusion episodes, cross‐matching and haemoglobin assessments

During a mean observation period of 4.7 (±0.7) years, patients had a mean of 13.7 (±3.2) transfusion episodes/year (see Supporting Information Table S2 and Figure S1) and 13.7 (±3.2) attendances for cross‐matching/patient/year (99% [10509/10592] of all cross‐matching tests were recorded on a different date to the transfusion]). The mean volume of blood transfused was 175.5 (±57.5) mL/kg/year (*n* = 84 patients with volume and weight recorded). Patients had a mean of 10.7 (±4.8) pre‐transfusion haemoglobin tests/year; the mean pre‐transfusion haemoglobin was 99.5 (±10.1) g/L during the observation period (*n* = 8473 tests).

### 3.3 Iron burden and iron chelation therapy

**FIGURE 2:**
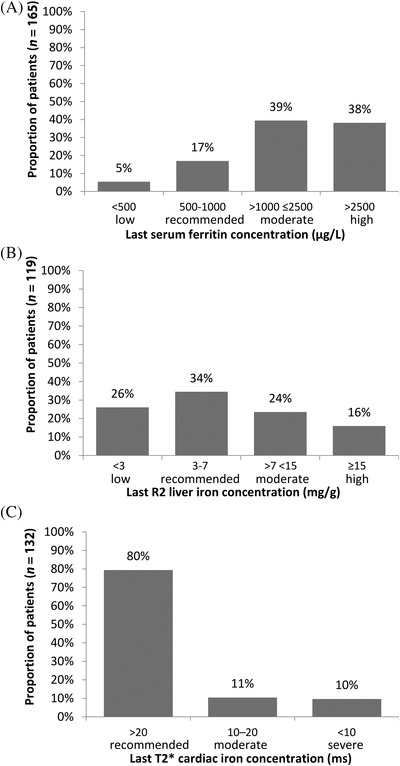
Iron burden at the last assessment during the observation period. Panel A: Last serum ferritin concentration. Panel B: Last R2 liver iron concentration. Panel C: Last T2* cardiac iron concentration. Cut‐off points for iron burden were based on UK Thalassaemia Society Standards [3]

Patients had a mean of 11.4 (±4.1) serum ferritin tests/year; the median serum ferritin concentration during the observation period was 1,544.0 (IQR 936.0–2640.0) μg/L (*n* = 8433 tests). The median serum ferritin at patients’ last documented assessment during the observation period was 1961.0 (IQR 1090.0–3003.0) μg/L; 5% had low serum ferritin (< 500 μg/L), 17% had recommended serum ferritin (500–1000 μg/L), 39% had moderate serum ferritin (>1000 to ≤2500 μg/L) and 38% had high serum ferritin (>2500 μg/L, Figure 2A).

Data on liver iron concentration (LIC) and cardiac iron assessments were available for *n* = 145 and n = 137 patients, respectively (assessed by any method). In the overall patient population, patients had a mean of 0.5 (±0.3) LIC/year (most commonly assessed by R2 magnetic resonance imaging [MRI]) and 0.4 (±0.3) cardiac iron assessments/year (most commonly T2* MRI). The median LIC at the last R2 assessment was 5.4 (IQR 2.9–11.6) mg/g (*n* = 119); 26% (31/119) had low LIC (<3 mg/g), 34% (41/119) had recommended LIC (3–7 mg/g), 24% (28/119) had moderate LIC (>7 to < 15 mg/g) and 16% (19/119) had high LIC (≥15 mg/g; Figure 2B). The median cardiac T2* at the last assessment was 30.3 (IQR 22.0–37.1) milliseconds (ms) (*n* = 132); 80% (105/132) had normal cardiac iron (>20 ms), 11% (14/132) had moderate cardiac iron (10–20 ms) and 10% (13/132) had severe cardiac iron (<10 ms, Figure 2C; 4/132 patients [3%] had very severe cardiac iron loading [<6 ms]).

In post hoc analyses, 44% of patients had average intervals of <2 years between LIC assessments and 29% had intervals of <2 years between cardiac iron assessments (Supplementary Figure S2). Intervals of <2 years were observed for 43% of patients with high LIC and 54% of patients with severe cardiac iron at the last assessment during the observation period (Supplementary Figure S3). The proportion of patients with an interval of <2 years between assessments of LIC and cardiac iron stratified by age at baseline are summarised in Supplementary Figure S4.

The median age at initiation of ICT was 2.9 (IQR 1.8–12.1) years (*n* = 91 patients with date of ICT initiation available). All patients received ≥1 iron chelator during the observation period, and 162 patients were receiving ICT at the end of the observation period (deferasirox: 58%; desferrioxamine: 14%; deferiprone: 7%; combination therapy: 21% [11% deferiprone/desferrioxamine, 5% deferasirox/deferiprone, 5% deferasirox/desferrioxamine]; Supplementary Table S3). Non‐adherence to ICT at any point during the observation period was documented in medical records for 25% of patients (23% of patients taking deferasirox, 12% of patients taking deferiprone, 8% of patients taking desferrioxamine and 3% of patients taking combination therapy). Non‐adherence was most commonly related to difficulties with compliance or incorrect dosing/frequency of dosing.

Overall, 28% of patients had at least one ICT‐related adverse event (AE) documented (Supplementary Table S3). Of the 132 patients prescribed deferasirox at any time during the observation period, 37 (28%) experienced a total of 64 deferasirox‐associated AEs, most commonly (each accounting for ≥5 deferasirox‐associated AE events) abdominal pain (30% of events) and hepatic impairment (14% of events); 50% of deferasirox‐associated AEs resulted in a change in ICT and 28% required treatment (Supplementary Tables S3 and S4). Of the 57 patients prescribed deferiprone, four (7%) experienced a total of nine different deferiprone‐associated AEs; 56% of these resulted in a change in ICT and 56% required treatment (Supplementary Tables S3 and S4). Of the 85 patients prescribed desferrioxamine, 11 (13%) experienced a total of 25 desferrioxamine‐associated AEs (each accounting for less than five desferrioxamine‐associated AE events); 16% of these resulted in a change in ICT and 48% required treatment (Supplementary Tables S3 and S4).

### 3.4 TDT‐related hospital attendances and admissions

In addition to attendances for blood transfusions and cross‐matching described above, ≥1 transfusion‐related outpatient attendance, day case admission, emergency department (ED) attendance, and inpatient admission were recorded for *n* = 159, *n* = 51, *n* = 57 and *n* = 41 patients, respectively (Supplementary Table S2). In the overall population, patients had a mean of 7.0 (±7.8) non‐transfusion‐related outpatient attendances/year (83% haematology [4545/5461 attendances]), a mean of 0.5 (±1.6) non‐transfusion‐related day case admissions/year, a mean of 0.2 (±0.4) ED attendances/year (30% resulting in admission) and a mean of 0.1 (±0.3) inpatient admissions/year (Supplementary Table S2). The mean length of stay for inpatient admissions was 5.6 (±6.2) days. Overall, patients had a mean of 7.8 (±8.1) non‐transfusion‐related hospital attendances or admissions/year. Fewer than 5% of non‐transfusion‐related outpatient attendances, day case admissions and inpatient admissions were to cardiology or endocrinology (Supplementary Table S2).

### 3.5 TDT treatment‐related complications and comorbidities newly recorded during the observation period

A mean of 0.4 (±0.3) iron overload complications were diagnosed/patient/year, most commonly (≥5 patients during the observation period) cardiac complications (11 [7%] patients, including one death due to cardiogenic shock), growth retardation (nine [5%] patients), hypothyroidism (seven [4%] patients), hepatic complications (seven [4%] patients) and diabetes (five [3%] patients). The comorbidities newly diagnosed during the observation period in ≥5 patients were vitamin D deficiency (16 [10%] patients) and recurrent urinary tract infections (six [4%] patients). No newly diagnosed infectious complications from blood transfusion were recorded.

### 3.6 Patient‐ and carer‐reported outcomes

**FIGURE 3:**
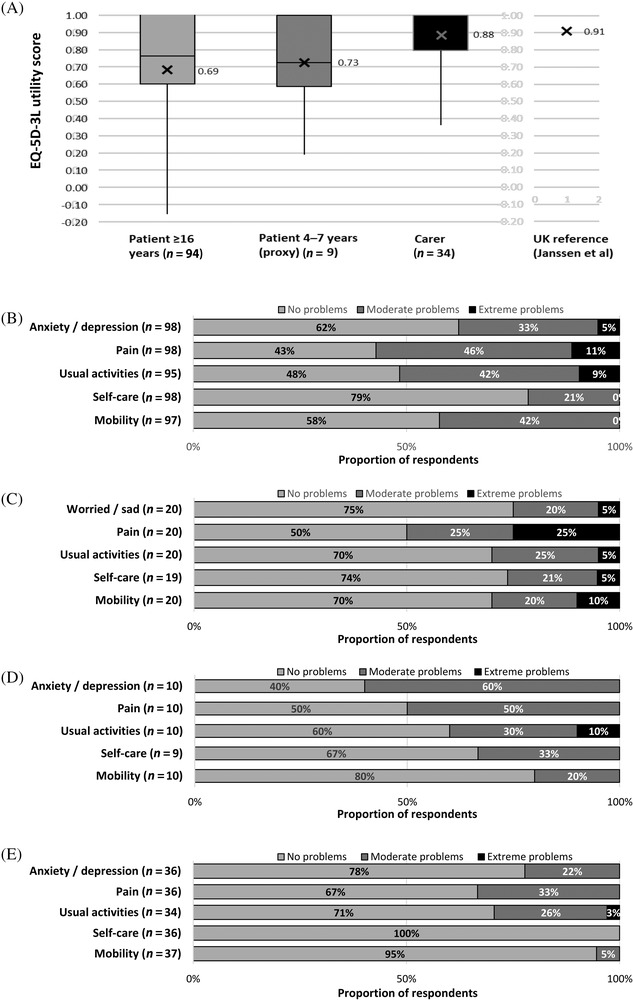
Patient and carer general health state assessed using age‐appropriate EQ‐5D‐3L questionnaires. Panel A: EQ‐5D‐3L utility score (*1* *equates to perfect health*, 0 *equates to death and negative values represent states worse than death* [14]) calculated using the UK value set [22]; there is currently no value set for the self‐completed EQ‐5D‐Y (8–15 years), therefore utility scores could not be calculated for this age group; the UK reference value was calculated from published age‐specific UK population norms weighted for the age‐distribution of the adult patients assuming the population norm for age 16–17 years was equivalent to age 18–24 years [43]; X indicates the mean. Panels B–E: Proportion of patients reporting problems according to EQ‐5D‐3L dimension level (B: adult patients ≥16 years; C: children 8–15 years; D: children 4–7 years [proxy]; E: carers). EQ‐5D‐3L domains were classified as level 1: no problems; level 2: moderate problems; level 3: extreme problems. (note: boxes represent the 25th percentile, 50th percentile [median] and 75th percentile of ranked scores; for the Carer utility score, the 50th and 75th percentile were 1.0 and consequently both are indicated by the top line of the box)

At least one patient‐reported outcome questionnaire was completed for 134/165 (81%) patients, with a median (IQR) time from diagnosis to questionnaire completion of 14.4 (8.0–23.4) years (*n* = 85 completed questionnaires and had date of diagnosis recorded). At least one carer‐reported outcome questionnaire was completed by 37 carers. In terms of general health status, an EQ‐5D‐3L utility score of 1 equates to perfect health and 0 equates to death. For the study population, the mean EQ‐5D‐3L utility score in patients aged ≥16 (*n* = 94) was 0.69 (±0.33), in patients aged 4–7 (proxy; *n* = 9) was 0.73 (±0.27) and in carers (*n* = 34) was 0.88 (±0.15); for reference, the age‐weighted utility score for a UK general adult population is 0.91; see Figure 3A (there is currently no value set for the self‐completed EQ‐5D‐Y [8–15 years], therefore no utility scores could be calculated for this age group). The problems reported by participants across the EQ‐5D‐3L individual QoL domains of anxiety/depression, pain, usual activities, self‐care and mobility are summarised in Figure 3B–E; moderate or extreme problems with pain were reported by ≥50% of patients in each age group (Figure 3B–D). The mean EQ‐5D‐3L VAS scores are summarised in Supplementary Table S5.

**FIGURE 4:**
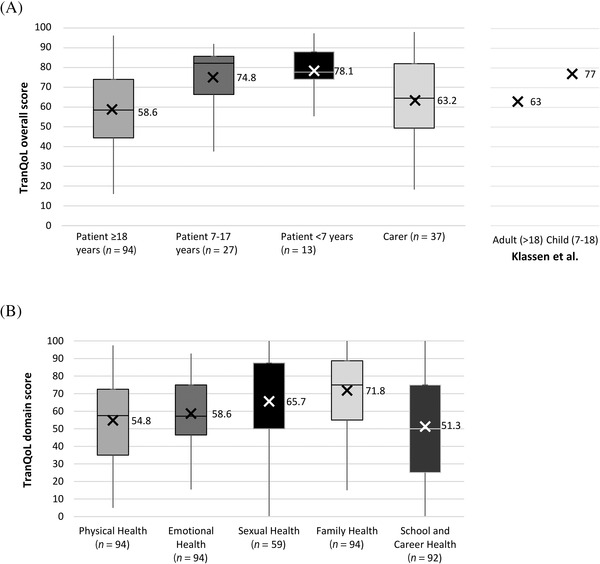
Patient and carer TDT‐related quality of life assessed using the TranQoL questionnaire. Panel A: Patient and carer TranQoL overall scores; X indicates the mean. Panel B: Patient TranQoL domain scores. *TranQoL score and individual domain scores range from 0 (worst thalassaemia‐related QoL) to 100 (best thalassaemia‐related QoL)*. X indicates the mean. (Note: boxes represent the 25th percentile, 50th percentile [median] and 75th percentile of ranked scores; for the Sexual Health domain scores the 25th and 50th percentiles were 50, and consequently both are indicated by the bottom line of the box)

For the TranQoL thalassaemia‐specific QoL instrument, a total TranQoL score of 100 represents the best and a score of 0 the worst disease‐related QoL for an individual living with TDT. The mean TranQoL score in patients aged ≥18 (*n* = 94) was 58.6 (±18.4), which represents notable impairment in QoL as related to living with TDT. Disease‐related QoL was also impacted in patients aged 7–17 (*n* = 27; mean score of 74.8 [±15.0]), in patients aged <7 (*n* = 13; proxy mean score of 78.1 [±12.7]) and in carers (*n* = 37; mean score of 63.2 [±21.4]) (Figure 4 and Supplementary Table S5).

**FIGURE 5:**
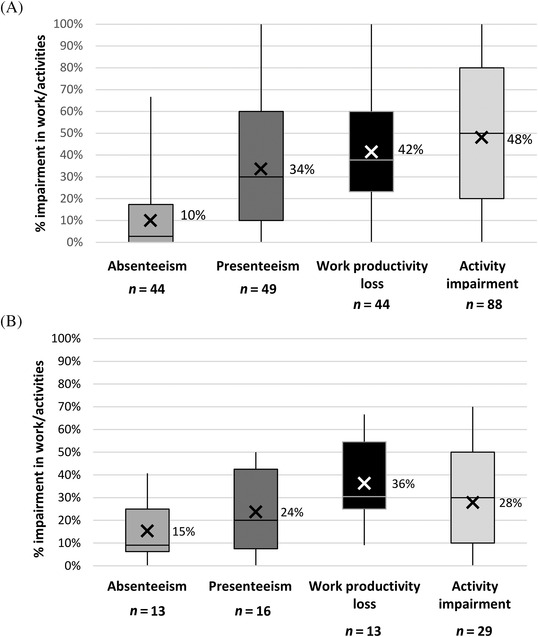
Patient and carer TDT‐related quality of life assessed using the WPAI questionnaire. Panel A: Adult patient domain scores for WPAI for specific health problems (WPAI‐SHP). Panel B: carer WPAI (WPAI‐CG) domain scores. The WPAI assesses the impact of disease on work productivity by evaluating absenteeism (the amount of work time missed), presenteeism (impaired work effectiveness [WPAI definition]) and overall work productivity loss (absenteeism + presenteeism); non‐work related activity impairment is also assessed [19, 20]. Domain scores are expressed as the percent impairment for work/activities. *Scores range between 0% (no impairment) to 100% (complete impairment)*. X indicates the mean. (note: boxes represent the 25th percentile, 50th percentile [median] and 75th percentile of ranked scores)

For the WPAI, scores are expressed as percent impairment, with higher scores indicating a greater degree of impairment. For patients ≥18 years, mean work productivity impairment was 42% (±28% [*n* = 44]) versus a typical working week, while the ability to do non‐work related activities was impaired by 48% (±32% [*n* = 88]). There was also a substantial impact of TDT on carers, with mean work productivity impairment of 36% (±20% [*n* = 13]) and non‐work related activity impairment of 28% (±23% [*n* = 29]) (Figure 5).

## 4 DISCUSSION

This study provides a contemporary (2018–2019) and comprehensive picture of the substantial impact of TDT on the daily lives of patients and their carers, and the significant burden on secondary healthcare resources in the UK. Patient demographics were consistent with the wider population of patients with thalassaemia [11, 23]. Based on recent data from England [24], we estimate that the study included approximately 15% of the UK population of patients with TDT. Comorbidities and TDT‐related complications were commonplace, with the latter representative of the spectrum of well‐documented complications [3, 11, 25, 26].

Patients had a mean of 13.7 blood transfusion episodes/year, consistent with a recent UK Hospital Episodes Statistics database study [11]. In addition, patients had an average of 13.7 cross‐match attendances and 7.8 other TDT‐related hospital attendances and admissions annually, equating to almost three TDT‐related appointments/month in total. The frequent hospital attendances and admissions have potential financial implications for patients and their families in terms of out‐of‐pocket expenses and impact on employment, as well as representing a considerable burden on healthcare resources [8, 27, 28, 29, 30].

Despite management in specialist centres, significant iron overload affected a subset of patients; 10% had severe cardiac iron loading, 16% high LIC and 38% high serum ferritin at the last assessment and one patient died due to cardiogenic shock. Chronic iron overload is associated with significant morbidity and mortality and, despite advances in ICT and monitoring, iron‐related cardiomyopathy remains the most frequent cause of death in patients with TDT [3, 9, 31, 32, 33]. Cardiac T2* iron was reported as the strongest predictor of cardiac iron‐related comorbidities, with 47% of patients with a cardiac T2* of < 6 ms developing heart failure within 12 months [32], emphasising the importance of regular iron overload monitoring. The UK Thalassaemia Society Standards recommend assessing cardiac iron every 6 months for patients with severe cardiac iron (<10 ms) and assessing LIC every 12 months for patients with moderate/high LIC (>7 mg/g) [3]. In post hoc analyses, we observed only 43% of patients with high LIC and 54% of patients with severe cardiac iron at the last recorded assessment had intervals of <2 years between assessments during the observation period. The study did not formally assess the underlying rationale for frequency of iron monitoring; however, inclusion of paediatric patients below the recommended age (7–10 years) for commencing MRI assessment of cardiac iron [3] may have contributed in part to the observation.

Although all patients received ICT at some point during the observation period, the proportion of patients with high iron burden suggests that iron overload was inadequately controlled by ICT in some patients. Consistent with this, 21% of patients were receiving combination ICT at the end of the observation period, most commonly deferiprone and desferrioxamine, which is recommended to improve cardiac iron deposition [3, 34, 35]. High iron burden may also partly reflect non‐adherence to ICT and at least one non‐adherence event was documented in 25% of patients; however, non‐adherence to desferrioxamine was lower than expected based on previously published patient‐reported and carer‐reported adherence [36]. The reason for this is unclear, but may partly reflect under‐reporting in routine clinical practice. Equally, patients on desferrioxamine may represent a motivated population who were managing complications and/or had good adherence with effective iron control and did not wish to switch to oral therapy. Non‐adherence to ICT has been suggested to limit the survival advantage afforded by full adherence [37] and may be influenced by age, difficulties with administration, the life‐long nature of treatment and the occurrence of side effects [36, 38]. In this context, AEs associated with ICT were common, and approximately 50% of all AEs required treatment, adding to the burden of routine management of TDT on patients and healthcare resources.

The adverse impact of TDT on QoL, reflecting the burden of frequent transfusions, monitoring and management of iron overload, treatment‐related AEs, TDT‐related comorbidities and other TDT‐related hospital attendances/admissions, has been described in various countries and using a variety of instruments [4, 5, 6, 7, 8, 39, 40, 41, 42]. This study provides further evidence of the substantial impact of TDT on a range of domains of daily living assessed using generic and disease‐specific instruments. The mean EQ‐5D‐3L utility score of 0.69 for adult patients suggests poorer QoL in comparison with the UK general population based on an age‐weighted mean of 0.91 estimated from published age‐specific UK population norms [43]. TranQoL is a recently developed TDT‐specific QoL questionnaire [17, 18], and, apart from the original studies, only studies using translated versions of TranQoL have been published with variable results [39, 40, 44], most likely reflecting differing social and healthcare environments. The mean TranQoL scores of adult patients and carers were broadly similar, suggesting a comparable impact of TDT on their QoL. Further studies of TranQoL are warranted to understand the factors influencing TDT‐related QoL in patients across the age spectrum. To the best of our knowledge, we show for the first time using the WPAI the marked impact of TDT on work productivity and general activity for both patients and carers. The impact on carers was similar to that reported for carers of chronically ill older people [21]. The impact on work productivity most likely reflects the frequency of routine TDT‐related daytime hospital attendances, particularly for cross‐matching and transfusions. Healthcare providers should examine how services can be adapted to ensure that transfusion‐related attendances are managed efficiently to minimise impacts on work and education. Taken together, the results of the present study emphasise the significant impact of TDT on the QoL of patients and their carers, with the extent to which children are affected by TDT across a range of domains of daily living particularly notable. Further studies are warranted to evaluate the impact of TDT on the daily lives of patients and their families over time.

Limitations of the study include the retrospective design and reliance on the quality of recording in source medical records. Furthermore, the generalisability to the wider population may be limited by the study's requirement for consent from living patients, and not all participants completing all questions.

## 5 CONCLUSIONS

These results offer important insights into the real‐world management and clinical status of patients with TDT in the UK, and underscore the significant burden of the condition on patients, carers and healthcare resources. In general, patients from the centres included appear to have been well managed; however, an important subset of patients suffer severe liver and cardiac iron loading, the latter being associated with significant mortality risk. The impact of the disease on the QoL of patients, in particular children, and their carers, should not be underestimated as evidenced by the range of domains of daily living that are affected.

## CONFLICT OF INTEREST

FS declares advisory board (silence therapeutics, Roche, Novartis, bluebird bio, Celgene), clinical safety committee (Abfero pharmaceuticals) and steering committee for trial (Celgene) involvement; PT declares advisory committee (Global Blood Therapeutics, Novartis, bluebird bio), data monitoring committee (Pfizer), clinical trial activity (Apopharma, Celgene, Global Blood Therapeutics, Novartis, Napp Pharma), investigator led funding (Kyowa Kirin Limited, bluebird bio) and speaker activity (Apopharma, Terumo plc); MV states advisory board activity for bluebird bio; SPancham declares advisory board (Celegene and Novartis) and sponsorship to attend educational meeting (Celegene); RW states nothing to declare; SPollard declares Novartis support to attend educational meetings, advisory board activity for bluebird bio; EC declares consultancy fees from Novartis; JK declares advisory boards with Celgene, Jazz and Novartis; AMC and JH are employees of pH Associates Ltd, doing business as OPEN Health; CP and MJ are employees of bluebird bio and own stock in the company; KR declares advisory boards for bluebird bio and Pfizer, Educational grant from Novartis. The study was funded by bluebird bio, Inc. bluebird bio, Inc. has a gene therapy for β thalassaemia currently licensed in the EU.

## AUTHOR CONTRIBUTIONS

FS, PT, MV, SPancham, RW, SPollard, EC, JK and KR were involved in the acquisition and interpretation of data, critical revision of the manuscript and approval of the final version for submission. AMC was involved in study design, interpretation of data, drafting and revision of the manuscript and approval of the final version for submission. JH was involved in study design, analysis and interpretation of data, critical revision of the manuscript and approval of the final version for submission. CP and MJ were involved in study design, interpretation of data, critical revision of the manuscript and approval of the final version for submission.

## DATA ACCESSIBILITY STATEMENT

**How or where can the data be obtained?**

Appropriately de‐identified patient‐level datasets and supporting documents may be shared following attainment of applicable marketing approvals and consistent with criteria established by bluebird bio and/or industry best practices to maintain the privacy of study participants. For more information please contact datasharing@bluebirdbio.com.

**When will data availability begin?**

Upon request following attainment of applicable marketing approvals.

## Supporting information



SUPPORTING INFORMATION
