# Supplementary material for: Routine management, healthcare resource use and patient and carer‐reported outcomes of patients with transfusion‐dependent β‐thalassaemia in the United Kingdom: A mixed methods observational study
Source: EJHaem. 2021 Sep 8;2(4):738–49. doi: 10.1002/jha2.282 (PMC9175788; doi:10.1002/jha2.282)

Supporting Information

Supplementary tables

Table S1. Questionnaires used to assess quality of life in patients and carers according to age category

		Patients			Carers
		**Adult**	**Child**	**Child proxy**	
**EQ-5D**	Age group	≥16 years	8–15 years	4–7 years	
	Version	EQ-5D-3L	EQ-5D-Y	EQ-5D-Y proxy†	EQ-5D-3L
**TranQoL**	Age group	≥18 years	7–17 years	<7 years	
	Version	TranQoL adult	TranQoL child	TranQoL proxy	TranQoL adult
**WPAI**	Age group	≥18 years	N/A	N/A	
	Version	WPAI-SHP	N/A	N/A	WPAI-CG

†Completed by carer on behalf of child aged 4–7 years (no version for those aged <4 years). N/A: questionnaire not applicable in children. TranQoL: transfusion dependent quality of life. WPAI-SHP: Work Productivity Activity Index for specific health problems. WPAI-CG: Work Productivity Activity Index for carers.

Table S2. Hospital attendances and admissions during the study observation period

Hospital attendances and admissions during the observation period	Patients with TDT (n=165)
Transfusion episodes/patient/year, mean (±SD)	13.7 (±3.2)
Transfusion cross-match attendances/patient/year, mean (±SD)	13.7 (±3.2)
Non-transfusion-related outpatient attendances/patient/year	7.0 (±7.8)
Total non-transfusion-related outpatient attendances during the observation period	n=5461 (in 159 patients)
Outpatient attendances by specialty, n (% of n=5461 attendances)	4545 (83%)
Haematology	143 (3%)
Ophthalmology	111 (2%)
Endocrinology	81 (1%)
Cardiology	76 (1%)
Audiology	2 (<1%)
	503 (9%)
Not known	
Other	
Non-transfusion-related day case admissions/patient/year	0.5 (±1.6)
Total non-transfusion-related day case admissions during the observation period	n=387 (in 51 patients)
Day case admissions by specialty, n (% of n=387 admissions)	
Haematology	232 (60%)
Radiology	98 (25%)
Ophthalmology	17 (4%)
Cardiology	9 (2%)
Gastroenterology	7 (2%)

Audiology	4 (1%)
Other	18 (5%)
Not known	2 (1%)
ED attendances/patient/year	0.2 (±0.4)
Total ED attendances during the observation period	n=144 (in 57 patients)
ED attendance only, n (% of n=144 attendances)	100 (69%)
ED attendance leading to admission, n (% of n=144 attendances)	44 (31%)
Inpatient admissions/patient/year	0.1 (±0.3)
Total inpatient admissions during the observation period	n=84 (in 41 patients)
Inpatient admissions by admission route/admitting specialty, n (% of n=84 admissions)	
ED	44 (52%)
Haematology	14 (17%)
Cardiology	2 (2%)
Endocrinology	2 (2%)
Other	18 (21%)
Not known	4 (5%)
Length of stay for inpatient admissions (days), mean (±SD)	5.6 (±6.2), n=84
Inpatient admissions via ED	*4.2 (±3.4), n=44*
Non-ED-related admissions	*7.2 (±8.0), n=40*
Total non-transfusion-related hospital attendances and admissions†/patient/year, mean (±SD)	7.8 (±8.1)

†Includes: all non-transfusion-related outpatient, Emergency Department (ED), day case and inpatient admissions. SD: standard deviation. TDT: transfusion-dependent β-thalassaemia.

Table S3. Routine management of TDT: iron chelation therapy

Iron burden and iron chelation therapy (ICT)	Patients with TDT (n=165)
Distribution of ICT during the observation period, n (% of patients)	
Deferasirox	132 (80%)
Deferiprone	57 (35%)
Desferrioxamine	85 (52%)
ICT ongoing at the end of the observation period, n (% of patients)	n=162
Deferasirox	94 (58%)
Deferiprone	11 (7%)
Desferrioxamine	23 (14%)
Combination therapy[†]	34 (21%)
Discontinued[‡]	3
Patients with documented non-adherence events, n (% of patients)	41 (25%)
Non-adherence to ICT during the observation period, n (% of patients taking chelator)	
Deferasirox	
Deferiprone	30/132 (23%)
Desferrioxamine	7/57 (12%)
Combination therapy	7/85 (8%)
	1/34 (3%)
**Adverse events (AEs) associated with ICT[§] during the observation period**	
Patients with ≥1 AE associated with ICT	46 (28%)
Distribution of AEs by type of chelator, number of AEs	
Deferasirox	64
Deferiprone	9
Desferrioxamine	25
Combination therapy	4
Patients with adverse events on ICT, n (% of patients taking chelator)	
Deferasirox	37/132 (28%)
Deferiprone	4/57 (7%)
Desferrioxamine	11/85 (13%)
Combination therapy	2/34 (6%)

AEs resulting in chelator therapy change, n (% of AEs associated with chelator)	
Deferasirox	32/64 (50%)
Deferiprone	5/9 (56%)
Desferrioxamine	4/25 (16%)
Combination therapy	0/4 (0%)
Chelator therapy change as a result of AEs, n (% of AEs requiring therapy change)	
Deferasirox discontinued	18 (56%)
Deferasirox dose reduced	14 (44%)
Deferiprone discontinued	4 (80%)
Deferiprone dose reduced	1 (20%)
Desferrioxamine discontinued	2 (50%)
Desferrioxamine dose reduced	1 (25%)
Desferrioxamine dose increased	1 (25%)
AEs requiring treatment, n (% of AEs associated with chelator)	
Deferasirox	18/64 (28%)
Deferiprone	5/9 (56%)
Desferrioxamine	12/25 (48%)
Combination therapy	1/4 (25%)

[†]Combination therapy: n=18/162 (11%) deferiprone + desferrioxamine, n=8/162 (5%) deferasirox + deferiprone, n=8/162 (5%) deferasirox + desferrioxamine. [‡]Reasons for discontinuation: n=1 patient deceased, n=1 pregnancy, n=1 infection. [c]Adverse events based on physician documented assessment. TDT: transfusion-dependent β-thalassaemia.

Table S4. Adverse events associated with use of iron chelation therapy

Adverse events associated with iron chelation therapy †	Number of adverse events	% of total adverse events for each chelator
**Deferasirox adverse events**		**% (n = 64)**
Abdominal pain	19	30%
Hepatic impairment	9	14%
Nausea	3	5%
Renal impairment	2	3%
Chromaturia	2	3%
Rash	2	3%
Pain or erythema at injection site	2	3%
Diarrhoea	1	2%
Increased liver transaminase	1	2%
Vomiting	1	2%
Other	22	34%
**Deferiprone adverse events**		**% (n = 9)**
Abdominal pain	1	11%
Arthralgia	1	11%
Hepatic impairment	1	11%
Rash	1	11%
Other	5	56%
**Desferrioxamine adverse events**		**% (n = 25)**
Skin reactions or swelling	4	16%
Injection site reaction	2	8%
Pain or erythema at injection site	2	8%
Abdominal pain	1	4%
Hepatic impairment	1	4%
Increased liver transaminase	1	4%
Itching	1	4%
Rapid heartbeat	1	4%
Other	12	48%

† Adverse events based on physician documented assessment.

Table S5. Patient and carer-reported outcomes questionnaires

Questionnaires	Patients	Carers
**EQ-5D-3L VAS scores,** mean (±SD)	≥16 years: 66.7 (±20.4), n=95	75.2 (±16.5), n=27
	8–15 years: 73.0 (±23.4), n=20	
	4–7 years‡: 79.2 (±20.4), n=10	
**WPAI (%),** mean (±SD)		
Absenteeism†	10% (±14), n=44	15% (±14), n=13
Presenteeism†	34% (±29), n=49	24% (±19), n=16
Work productivity†	42% (±28), n=44	36% (±20), n=13
Activity impairment	48% (±32), n=88	28% (±23), n=29
**TranQoL,** mean (±SD)	≥18 years: 58.6 (±18.4), n=94	63.2 (±21.4), n=37
	7–17 years: 74.8 (±15.0), n=27	
	<7 years‡: 78.1 (±12.7), n=13	

†Only patients in employment. ‡Proxy completed by carer of child in relation to the child. SD: standard deviation. TranQoL: transfusion dependent quality of life. VAS: visual analogue scale. WPAI: Work Productivity Activity Index.

Supplementary figures

Figure S1. Red blood cell transfusion episodes per year

The number of blood transfusion episodes/year ranged from 6.0 to 31.8.

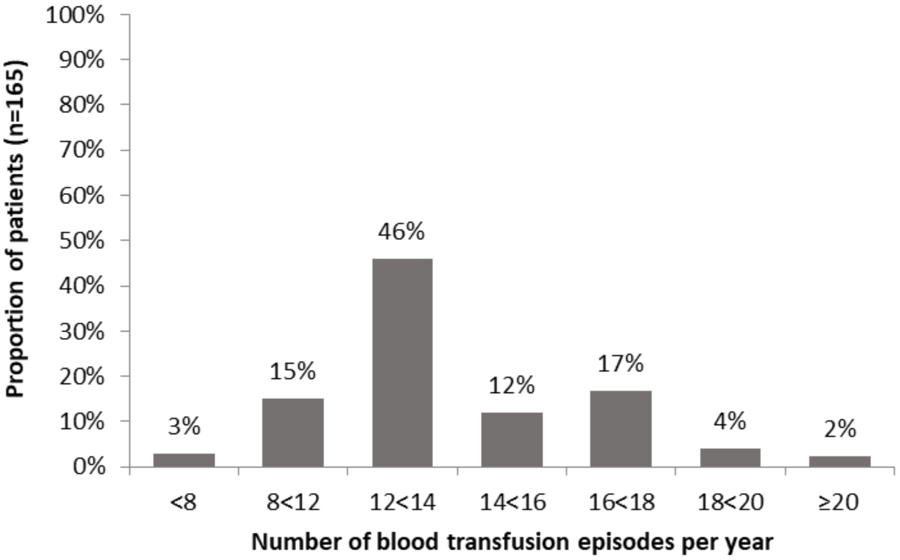

Figure S2. Interval between liver and cardiac iron assessments during the observation period. Panel A: Average interval between liver iron assessments (any protocol). **Panel B:** Average interval between cardiac iron assessments (any protocol). N/A: not applicable - patients did not have any assessment recorded during the observation period

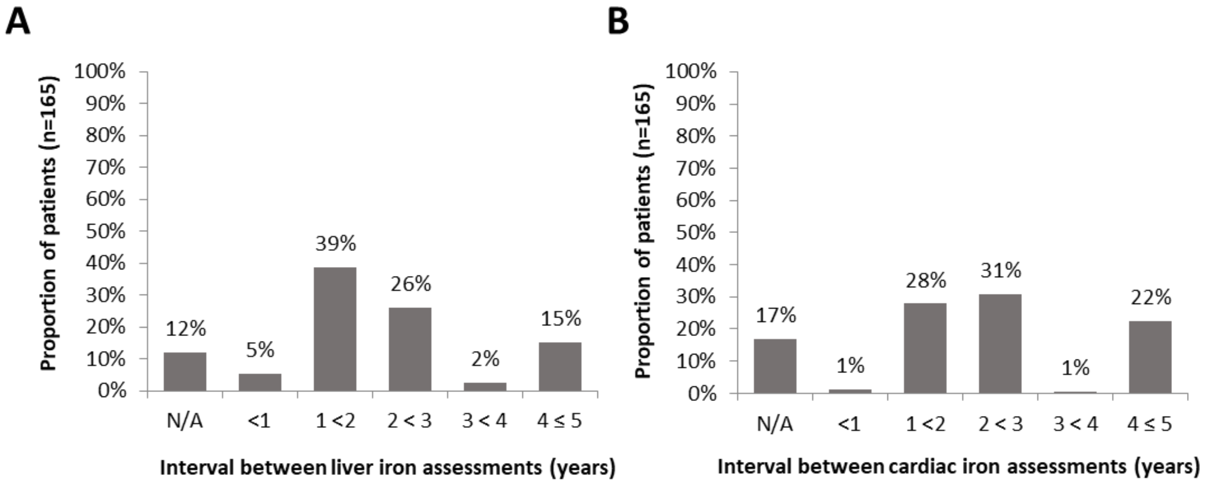

Figure S3. Interval between liver and cardiac iron assessments, stratified by concentration at the last assessment during the observation period. Panel A: Average interval between liver iron assessments (any method of assessment reported in mg/g). **Panel B:** Average interval between cardiac iron assessments (any method of assessment reported in ms).

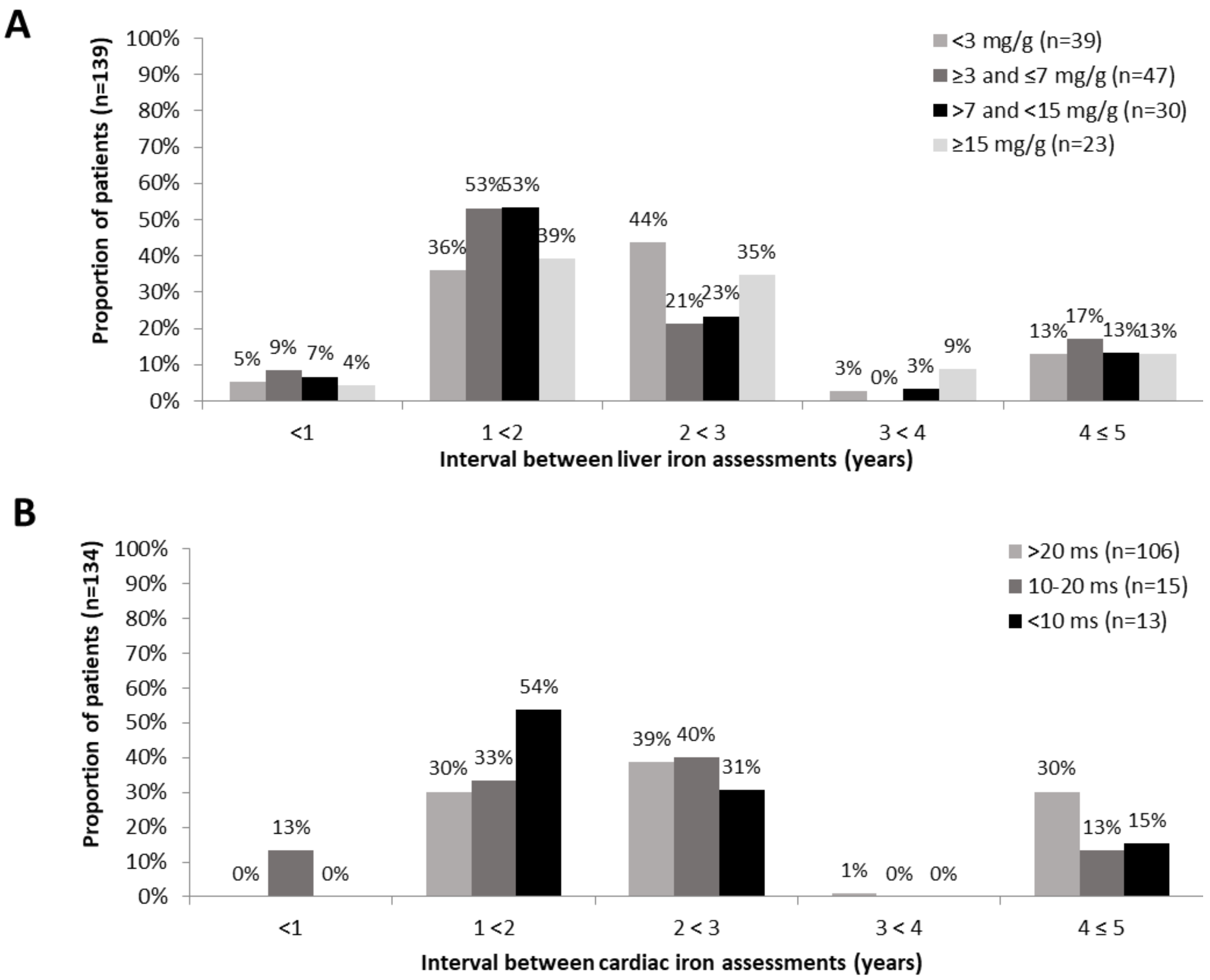

Figure S4. Interval between liver and cardiac iron assessments, stratified by age at baseline. Panel A: Average interval between liver iron assessments (any method of assessment). **Panel B:** Average interval between cardiac iron assessments (any method of assessment). N/A: not applicable - patients did not have any assessment recorded during the observation period

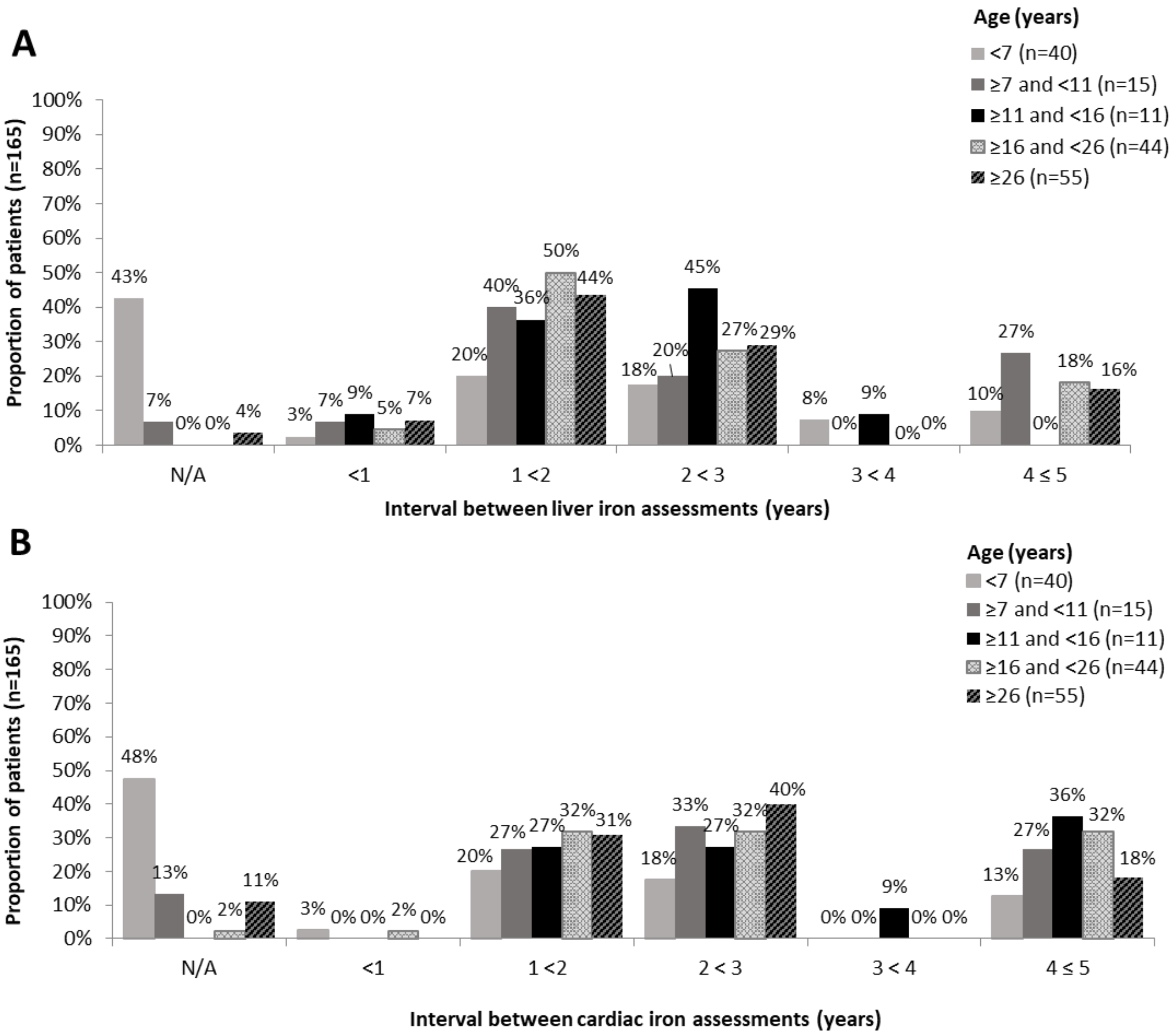

Supplement: Supplementary file 1 — SUPPORTING INFORMATION [file JHA2-2-738-s001.pdf]
